# Cu(I/II) Metal–Organic Frameworks Incorporated Nanofiltration Membranes for Organic Solvent Separation

**DOI:** 10.3390/membranes10110313

**Published:** 2020-10-29

**Authors:** Lakshmeesha Upadhyaya, Yu-Hsuan Chiao, S. Ranil Wickramasinghe, Xianghong Qian

**Affiliations:** 1 Department of Biomedical Engineering, University of Arkansas, Fayetteville, AR 72701, USA; 2 King Abdullah University of Science and Technology (KAUST), Biological and Environmental Science and Engineering Division (BESE), Advanced Membranes and Porous Materials Center (AMPM), Thuwal 23955-6900, Saudi Arabia; lakshmeesha.upadhyaya@kaust.edu.sa; 3 Ralph E Martin Department of Chemical Engineering, University of Arkansas, Fayetteville, AR 72701, USA; ychiao@uark.edu

**Keywords:** metal–organic frameworks (MOFs), mixed-matrix membranes, solvent-resistant nanofiltration, toluene-heptane separation, separation factor

## Abstract

Copper-based metal–organic frameworks (MOFs) with different oxidation states and near-uniform particle sizes have been successfully synthesized. Mixed-matrix polyimide membranes incorporating 0.1–7 wt% of Cu(II) benzene-1,2,5-tricarboxylic acid (Cu(II)BTC), Cu(I/II)BTC and Cu(I) 1,2-ethanedisulfonic acid (EDS) (Cu(I)EDS) MOFs were fabricated via non-solvent-induced phase inversion process. These membranes are found to be solvent resistant and mechanically stable. Liquid phase nanofiltration experiments were performed to separate toluene from n-heptane at room temperature. These membranes demonstrate preferential adsorption and permeation of the aromatic toluene over aliphatic n-heptane. The amount of MOF particles incorporated, the oxidation state of the Cu ion and membrane, and barrier layer thickness have a significant impact on the separation factor. Toluene/heptane separation factor at 1.47, 1.67 and 1.79 can be obtained for membranes incorporating 7 wt% Cu(II)BTC, Cu(I/II)BTC and Cu(I)EDS respectively at room temperature.

## 1. Introduction

The aliphatic and aromatic compound separation is one of the significant challenges faced by the petrochemical industry, especially in the refining field [1,2,3,4,5,6]. The process of separation is required due to the strict regulatory prerequisite by different governing bodies during the production of cyclohexane, the removal of sulphur from gasoline as well as in the naphtha reforming field [1,2,3,4,5,6]. It is more challenging to separate the aliphatic and aromatic compounds with closer boiling points, similar physical and chemical properties. Current industrial applications primarily depend on the complex azeotropic and extractive distillation procedures that require significant capital investment as well as energy input [1].

Membrane technology has become an alternative to the traditional distillation processes due to its ease of operation, energy efficiency, eco-friendliness and easy scale up [7,8,9,10]. Pervaporation is the most common approach used in the separation of aromatic/aliphatic compounds such as toluene and n-heptane based on their differences in solubility and diffusion coefficient of the vapor phase molecules across the membrane materials. The common pervaporation membrane materials used for the separation of n-heptane and toluene polymers are polyurethanes, polyetherimides, poly (vinyl chloride) (PVC), polyacrylates and their copolymers [2,7,8,9,10,11,12,13,14,15,16,17,18,19,20,21,22,23,24,25]. These membranes demonstrated relative high selectivity. However, one of the drawbacks is its low flux. Moreover, these membranes are not stable when harsh conditions are applied. Another major issue associated with these membranes for the pervaporation application of aliphatic-aromatic separation is their high degree of swelling which adversely affects the separation factor due to the subsequent increase in the fractional free volume (FFV) of these polymeric materials.

An ideal industrial membrane process to separate toluene from n-heptane should have high selectivity, high permeance and is economically competitive. Since the boiling points under atmospheric condition for toluene and n-heptane are 100.8 and 98.4 °C and the kinetic diameters are 5.1 and 4.9 Å, respectively, the physical properties of these two molecules are very similar to each other and hard to distinguish. However, since toluene is aromatic and has very different chemical properties from n-heptane, separation of these two compounds based on their chemical property differences is possible. It is known that aromatic compounds possessing conjugated π bonds. These conjugated π bonds are known for π–π stacking interaction as well as for cation- π interaction [26,27]. These two types of interactions are ubiquitous in nature contributing to many specific biological conformations and self-assembly of polymers and macromolecules [10,28]. In particular, cation- π interaction commonly exists in metal–organic frameworks (MOFs) and is critical for the structure, properties, and stability of the formed MOFs [29,30].

In order to separate toluene from n-heptane using membrane-based liquid separation processes, it is essential that a tight nanofiltration membrane with affinity to the aromatic compounds should be used. In addition, this membrane should resist to swelling from organic solvents in order not to compromise the separation performance. A number of approaches have been used to enhance the affinity between toluene and the membrane materials as well as reduce membrane swelling. Most of these approaches include surface modification of commercial membranes [11,18,21,31,32] as well as incorporating inorganic nanoparticles in the polymer matrix to form mixed matrix membranes (MMMs) [18,21,33,34].

A previous study [13] used inorganic nanoclays as fillers embedded in the PVC membranes for the separation of toluene and n-heptane. With 10 wt% loading of the nanoclays, the mixed matrix membranes exhibited approximately 300% increase in flux with a separation factor of 6 at 50 °C compared to PVC membranes without the nanoclay fillers operated at the same condition. Another work [35] fabricated the polyelectrolyte membranes consisting of methyl methacrylate and methacrylic acid. They further incorporated Co^2+^ to cross-link the polymers. The preferred cation-π interaction between the Co^2+^ ions and the aromatic π electrons of the toluene leads to a preferential selection of the toluene in the permeate during pervaporation. The flux and separation factor achieved are 2100 g·m^−2^·h^−1^ and 3.5 respectively at 85 °C. Earlier work [24] used Fe^3+^ and Co^2+^ ions to cross-link methacrylic acid and methyl methacrylate forming a hybrid membrane for the separation of benzene and cyclohexane based on the similar cation-π interaction between the cations and benzene molecules. Novel graphene oxide [12] and activated carbon [33] incorporated in the PVC membranes have also been used for the aliphatic and aromatic compound separations due to the presence of π−π interactions between the aromatic carbon and toluene. Wang et al. [36] used membranes fabricated with hyperbranched polymers (Boltorn W3000) grafted with unsaturated fatty acid and polyethene glycol (PEG) to separate toluene from n-heptane.

Metal–organic frameworks (MOFs) also called porous coordination networks are a new class of porous crystalline organic-inorganic hybrid materials. They have been extensively explored for many applications including gas storage, gas separation, catalysis, sensing and light harvesting [29,30,37,38,39,40,41]. MOF materials have been targeted predominantly due to their chemical compatibility, mechanical stability and their highly porous nature [42]. Previous work largely demonstrated their application for gas separations as well as for the separation of alcohol/water with pervaporation. Zhang et al. [16] used the Cu(II) benzene-1,2,5-tricarboxylic acid (Cu(II)BTC) incorporated in the PVC polymer matrix to prepare the nanohybrid membranes on the ceramic tubular substrate for the separation of toluene/n-heptane by pervaporation at 40 °C. Although still too low, the flux increased tenfold from 0.016 to 150 g·m^−2^·h^−1^ with the addition of MOFs in the PVC polymeric matrix. A separation factor of 17.9 was achieved for hybrid membranes compared to 8.9 for the PVC-only membranes. The affinity interaction between the cation and aromatic compounds due to the hybridization between the unoccupied *d* orbitals of the transition metal ions and the occupied *p* orbitals of aromatic materials plays a significant role in the separation of toluene over aliphatic compounds. In addition, the aromatic organic linker in the Cu(II)BTC also contributes to the affinity interaction due to the π–π interaction. Earlier studies [43,44] also showed that linkers with -COOH, -OH, -NH_2_ and -SO_3_H groups also play an essential role in the adsorption of toluene molecules in MOFs embedded in the polymer matrix resulting from their relative strong dipolar interactions.

Previous investigations focused on the pervaporation process for the separation of toluene and n-haptane, which required an elevated temperature and high energy input. Here, solvent-resistant nanofiltration (SRNF) membranes were fabricated and operated at room temperature to separate toluene from n-haptane. These SRNF membranes take advantage of the cation–π interaction between the Cu^2+/^Cu^+^ ions and the aromatic benzene ring on the toluene to achieve the separation between the two molecules. In order for the membranes to operate in the nanofiltration range, the particle size and particle size distribution of the MOFs incorporated in the polymer matrix need to be carefully controlled. Here near-uniform particle size with 25 nanometer (nm) of the Cu-based MOFs were synthesized by controlling the concentration ratio of the organic linker and a modulator following a protocol in the literature [45]. Moreover, MOFs with different Cu oxidation states have been synthesized and investigated for the separation performances using toluene and n-heptane solvent mixtures. MOFs with different oxidation state of the Cu ion were synthesized by reducing Cu(II)BTC using hydroquinone resulting in the formation of Cu(I/II)BTC with mixed Cu^+^/Cu^2+^ [46]. In addition, reduced Cu(I) MOF using linker 1,2-ethanedisulfonic acid (EDS) was fabricated with a similar particle size of about 25 nm [47]. Since cation–π interaction results from the coupling of the *d* orbitals of the cation and the *p* orbitals of the aromatic compound, different oxidation states of the Cu ion will lead to different couplings thereby their interaction energies with the aromatic compound. It is expected that the oxidation state of the MOF materials incorporated will affect the separation performance of the mixed matrix membranes fabricated. In addition, separation selectivity is also expected to be dependent on the type and amounts of MOFs incorporated and corresponding membranes formed.

Our work is the first of its kind in separating toluene from n-heptane in the liquid phase using a room temperature solvent-resistant nanofiltration process, unlike previous studies performed using a pervaporation process. Moreover, mixed matrix membranes incorporating MOFs with different oxidation states of the Cu ions were fabricated and compared for their separation performances. Significant insights were obtained on the selectivity of toluene over n-heptane using these hybrid membranes.

## 2. Experimental

### 2.1. Reagents

Copper(II) acetate monohydrate (98%) (Alfa Aesar, Ward hill, MA, USA), dodecanoic acid (DA, 98%) (Alfa Aesar, Hesham, LA, USA), benzene-1,2,5-tricarboxylic acid (BTC, 98%) and hydroquinone (Beantown Chemicals, Hudson, NH, USA), 1,2-ethanedisulfonic acid (EDS) dihydrate (TCI, Portland, OR, USA), 4,4′ bipyridine (99%) (Beantown Chemicals, Hudson, NH, USA), hydroquinone were used as received for synthesis. For MOF synthesis, 1-butanol was purchased from Alfa Aesar (Ward Hill MA, USA). For MOF purification, methanol and ethanol were purchased from VWR Analytical (Radnor, PA, USA). The polyimide (P84) powder was obtained from HP Polymer GmBH (Lenzing, Austria) and the cross-linking agent 1,6 diamino hexane (98%) is from Alfa Aesar (Ward Hill, MA, USA). For membrane synthesis, dimethylformamide (DMF) 1,4 dioxane and isopropanol (IPA) were obtained from VWR Analytical (Radnor, PA, USA). Polyethylene glycol (PEG, 400) for membrane pore conditioning was purchased from TCI (Portland, OR, USA). For solvent filtration and detection in HPLC, n-heptane, toluene, HPLC grade acetonitrile were purchased from MilliporeSigma Corporation (Billerica, MA, USA).

### 2.2. Synthesis of Cu(II)BTC

A typical synthesis of Cu(II)BTC was conducted as follows: copper acetate monohydrate (78.6 mg, 0.39 mmol) and dodecanoic acid (2.2 g, 0.011 mol) were first dissolved in 20 mL of butanol at 60 °C. The solution was then cooled down to room temperature and transferred into a Teflon cell (50 mL capacity) containing BTC (46.2 mg, 0.22 mmol). The Teflon cell was then sealed in a stainless-steel reactor (Toption instruments, HeCheng, China) and placed in a preheated (140 °C) fluidized sand bath (Techne^®^, Cole-Parmer, Staffordshire, Great Britain). After the reaction, the stainless-steel reactor was removed from the sand bath and cooled naturally. The resulting blue powder was separated by centrifugation and washed with ethanol for three times. The powder was then dried under vacuum overnight at 40 °C.

### 2.3. Reduction of Cu(II)BTC to Cu(I)BTC

Following a protocol in the literature [46] for the reduction of Cu(II)BTC to Cu(I)BTC, 100 mg of Cu(II)BTC and 1.0 g of hydroquinone were mixed with 10 mL of deionized (DI) water in the Teflon cell. After the reducing agent hydroquinone was dissolved in water completely, the cell was then sealed in the stainless-steel reactor and transferred to the pretreated sand bath at 150 °C. After 16 h of reaction, the black solid was collected by centrifugation and washed with methanol twice. The dark-coloured solid was then vacuum-dried overnight and stored in an airtight brown bottle to avoid oxidation.

### 2.4. Direct Synthesis of Cu(I)EDS

The direct synthesis of Cu(I)EDS was carried out by modifying an existing procedure [47]. More specifically, copper acetate monohydrate (270 mg, 1.35 mmol), 1,2-ethanedisulfonic acid (EDS, 290 mg, 1.52 mmol) and 4,4′ bipyridine (210 mg, 1.34 mmol) were first mixed with 10 mL of water. The solution was then stirred at room temperature for 30 min and transferred to the Teflon cell. The Teflon cell was subsequently sealed and placed in the stainless-steel reactor that was then placed in the preheated sand bath at 150 °C. After the reaction was carried out for four days, the reactor was cooled down naturally. The pH of the solution changed from the initial 4.2 to 3.1 after the reaction. The yellow-brown crystals were then filtered and washed with water and acetone mixture for 2–3 times. The crystals were then dried overnight in a vacuum oven.

### 2.5. Synthesis of MMMs

The polyimide (PI) polymer was selected as the polymer matrix since it has shown to be resistant to organic solvents. A total of 20 wt% of PI was dissolved in a mixed solvent consisting of 1:1 *v/v* DMF and 1,4-dioxane. To avoid the structural deformity, 20 wt% of polymer concentration was selected. Higher polymer concentration leads to increased viscosity that affects the phase inversion process significantly [48]. The resulting solution was stirred overnight to reach a homogeneous solution. MOF in different dosage (0.1–7 wt%) was added step by step to the polymer solution. After adding all the pre-determined amount of MOF, the solution was sonicated for 30 min and stirred for 90 min. The sonication-stirring cycle was repeated 4 to 5 times in order to disperse the MOF uniformly. After mixing the casting solution for two days, the polymer solution was allowed to stand overnight to remove the air bubbles trapped. The polymer solution was then cast on a polyester nonwoven support using a casting knife with a thickness of 200 µm. Immediately after casting, the membrane was transferred to a water bath at 25 °C to facilitate the non-solvent-induced phase separation and was kept in water for 1 h. Thereafter the membrane was transferred to a solvent bath containing isopropanol for 15 min to remove any residual water and DMF. The support was subsequently cross-linked by immersing the membrane in a cross-linking solution of IPA containing 50 g/L 1,6 diamino hexane for 16 h at room temperature. After that, the membrane was washed with isopropanol 2 to 4 times to remove any residual cross-linking agent. The fabricated membrane was then transferred to polyethylene glycol (PEG, MW 400) solution with 3:2 PEG/IPA to avoid pore collapsing during the filtration experiments. The membrane was soaked in the solution overnight (12 h). After conditioning, the membrane was immediately used for filtration after wiping it with a tissue paper to remove any residual liquid. For each condition, three sets of membranes were fabricated.

### 2.6. Characterizations

Measurement of the specific surface area of fabricated MOF was carried out using Micrometrics Tristar (Norcross, GA, USA) with N_2_ at 77.4 K. The specific surface area was calculated based on Brunauer–Emmett–Teller (BET) method [49]. Thermogravimetric analysis was carried out with Mettler Toledo TGA/SDT (Columbus, OH, USA) A851c LF/1100 °C with MT 5 balance and Pt−Pt/Rh 30% thermo element sensors. The analysis was performed from 25 °C to 800 °C with a heating rate of 10 °C/min.

The composition of the reduced MOF was detected by Versaprobe X-ray photoelectron spectroscopy (XPS, K-alpha; Thermo Scientific, Waltham, MA, USA). The synthesized MOFs were placed on double-sided tape on a glass substrate, and the data were collected using a monochromated Al Kα (h*v* = 1436.6 eV) X-ray source. Survey scans (0–1350 eV) were taken for each sample at a pass energy of 200 eV for every 1 eV. High-resolution scans were taken at pass energy (constant analyzer energy mode) of 50 eV for every 0.1 eV. The relative abundance of elements was determined using *Avantage* software, which integrated and compared the areas under the carbon and oxygen photoemission peaks.

The top surface and cross-section of the membranes were characterized by Quanta FEG-250 scanning electron microscope (SEM) (Thermo Fisher Scientific, Waltham, MA, USA). It is an environmental SEM for high-resolution imaging and compositional analysis using energy-dispersive X-ray microanalysis (EDS).

### 2.7. Adsorption Studies

The PI polymer solutions containing 0.1 wt% of three different MOFs were prepared. The casted membranes were subsequently transferred to a sealed glass vessel containing toluene and heptane mixtures (10 mL, ~6.8 g). The amount of toluene in the mixture varied from 0 to 20 mg for the equivalent toluene concentrations of 0–2 mg/mL. The vessel was stirred at 130 rpm. The toluene concentration was measured at every few hours up to 28 h. The analysis indicated that equilibrium was reached at around 23 to 24 h. For different loadings of MOF in the membrane, samples were collected at 24 h to determine the equilibrium concentration using HPLC. Only the highest toluene concentration of 2 mg/mL in the solvent mixture was used. Langmuir isotherms [50] were used to fit the experimental data. Langmuir adsorption isotherm describes layer-by-layer adsorption. Equation (1) below represents the Langmuir isotherm where Q_e_ is the amount of toluene adsorbed per gram of adsorbent at equilibrium in mg/g, Q_sat_ is the Langmuir maximum adsorption capacity in mg/g, C_e_ is the concentration of adsorbate at equilibrium in mg/g, K_L_ is the Langmuir constant in g/mg.

(1) $$\frac{C_{e}}{Q_{e}}=\frac{1}{Q_{sat}‗K_{L}}+\frac{C_{e}}{Q_{sat}}$$

### 2.8. Membrane Performance

Solvent filtration experiments were carried out using HP4750 Sterlitech stainless steel stirred cell (Sterlitech Corporation, Kent, WA, USA) with 14.6 cm^2^ membrane active area for filtration. The upstream side of the cell was connected to nitrogen cylinder through pressure valve. The downstream side was connected to a closed glass vessel through small stainless-steel tubing. The glass vessel was placed on weighing balance online with the computer system to record the weight of the permeate.

The toluene composition in the feed was kept at 12, 31 and 58 mol%. For calculation of separation factor, molar fractions were considered. The pressure was increased in a stepwise fashion with 10 min interval for every 50 psi increase in pressure. After 30 min, the pressure was increased to 180 psi. Based on the concentrations of MOF and cross-linking agent, the permeate stream took 35 min to 6 h to come out after applying the 180 psi of pressure at the upstream side. The flux reached a constant value very quickly after which permeate steam composition was analyzed. For error bars, three different sets of experiments were carried out in which 2 membranes has been taken from the same batch of fabricated membranes and one from a new batch of fabricated membranes to show the reproducibility for membrane fabrication steps.

The toluene concentration in permeate stream was measured by high-performance liquid chromatography (HPLC, Agilent Technologies-1260, infinity quaternary LC, Santa Clara, CA, USA). The UV-vis detector was set at wavelength 254 nm. Phenomenex C-18 column (Luna^®^) with bon pack 5 µm (250 × 4.6 mm) analytical column was used. 100% acetonitrile was used as mobile phase at a 0.45 mL/min flow rate. The standard curve is shown in Figure S1 of the supplementary document.

The solvent flux (J) [51] was calculated as the permeate volume (V) divided by the membrane area (A) and filtration time (t)

(2) J=V/(A×t)

The separation factor (α) [51] is a measure of the ability of the membrane to separate the different components in a feed under a specific condition and is calculated using Equation (3) below,

(3) $$\alpha =\left(Y_{Tp}/Y_{Hp}\right)/\left(Y_{Tf}/Y_{Hf}\right)\;$$

where Y is molar fraction, T and H represent toluene and n-heptane respectively, f and p denote feed and permeate streams respectively.

The degree of swelling [4] was calculated based on membrane weight before filtration (W_BF_) and membrane weight after filtration (W_AF_) and is calculated using Equation (4) below:

(4) $$\mathrm{Degree}\;\mathrm{of}\;\mathrm{swelling}=\left(W_{\mathrm{AF}}-W_{\mathrm{BF}}\right)/W_{\mathrm{BF}}$$

## 3. Results and Discussions

### 3.1. Synthesis and Characterization of MOFs

**Figure 1:**
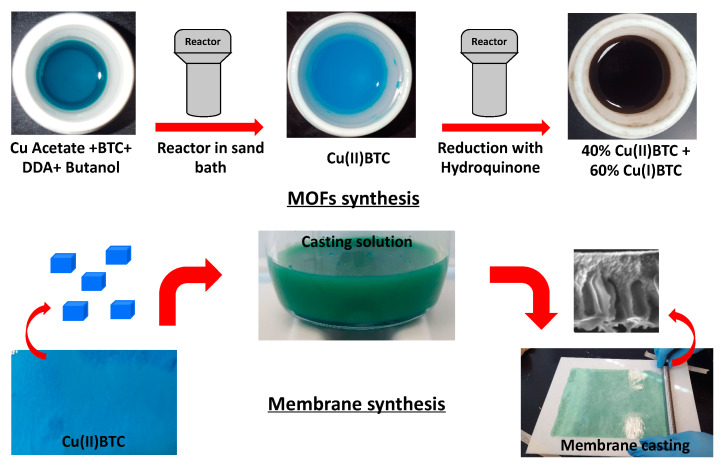
Schematic representation of fabrication of metal-organic frameworks (MOFs) and mixed-matrix membranes (MMMs).

Previous studies [30,40] show that Cu(II)BTC can be successfully synthesized using copper nitrate and BTC as precursors in water-ethanol or DMF solvents. This synthesis method requires high temperature and extended reaction time. Another primary concern over this approach is that there is no control over the size of the Cu(II)BTC synthesized. A different strategy as shown in Figure 1 was used in order to control the size of the Cu(II)BTC synthesized [45]. Here a modulator (dodecanoic acid, DA) was used in the precursor solution containing copper acetate and BTC in butanol solvent. It was suggested that the use of copper acetate speeds up the reaction due to the formation of copper dimers. The use of butanol solvent increases the viscosity of the reaction mixture which affects the reaction kinetics thereby the size and crystallinity of the synthesized MOFs. The concentration ratio of dodecanoic acid to BTC was kept at 50, which resulted in the formation of cubic-like crystals compared to other fused structures at different ratios. The use of modulator reduces the surface energy of the crystals which in turn leads to the growth of smaller size crystals.

**Figure 2:**
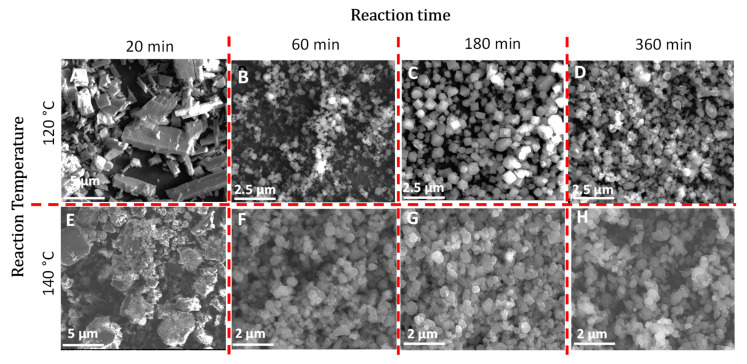
Scanning Electron Microscopy (SEM) images of Cu(II)BTC fabricated using dodecanoic acid as a modulator at various reaction temperature and time. Synthesized MOFs at 120 °C are shown in images A-D representing 20, 60, 180 and 360 min of reaction time respectively. The corresponding times at 140 °C are shown in images E-H.

Here a stainless-steel reactor was used with the synthesis conducted for 20, 60, 180 and 360 min at 120 °C and 140 °C respectively. Figure 2 shows the SEM images of fabricated MOFs at different conditions. It can be seen that the MOF crystals after 180 min of reaction appear to be uniform at both temperatures. Both autoclave and sand bath were utilized to heat the reactors. During the autoclave method, larger irregular MOF crystals evolved, as shown in Figure S2 in the supplementary document. It appears that sand bath was superior over autoclave due to the vibration of the hot sand in the sand bath which leads to a better mixing of the reactants.

**Figure 3:**
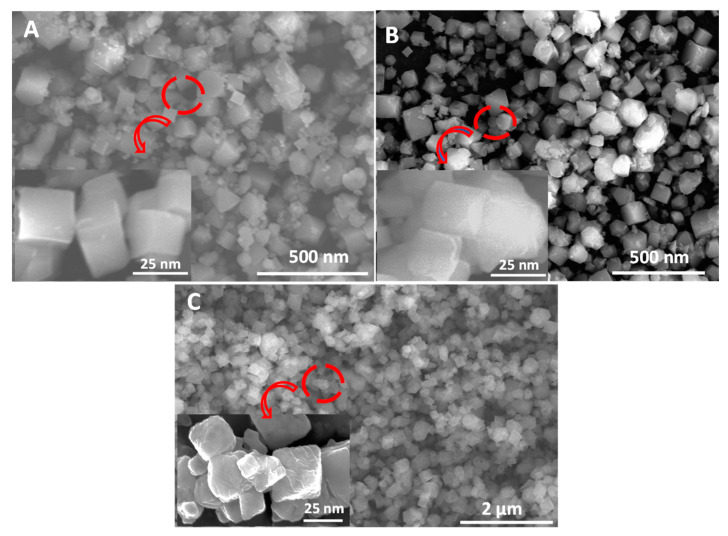
SEM images of MOFs synthesized using a stainless steel reactor (**A**) Cu(II)BTC; (**B**) Cu(I/II)BTC reduced from Cu(II)BTC; and (**C**) direct synthesis of Cu(I)EDS.

Stainless-steel reactor under sand bath has also been used for the synthesis of Cu(I)EDS as well as for the reduction of Cu(II)BTC which was previously synthesized as a starting material with water as a solvent. The specific conditions for both reactions have been described previously. Figure 3 shows the SEM of Cu(II)BTC, Cu(I/II)BTC reduced from Cu(II)BTC as well as directly synthesized Cu(I)EDS.

Figure S3 in the supplementary document shows the particle size distribution of MOFs varying around 25 nm. The N_2_ adsorption and desorption studies were carried out to further characterize the properties of fabricated MOFs as shown in Table 1. All three MOFs show a similar particle size in the range of 22–25 nm with specific surface areas between 980–1120 m^2^g^−1^. However, the size of Cu(II)BTC is the largest among the three with Cu(I)EDS the smallest. The BET surface area exhibits an opposite trend.

**Figure 4:**
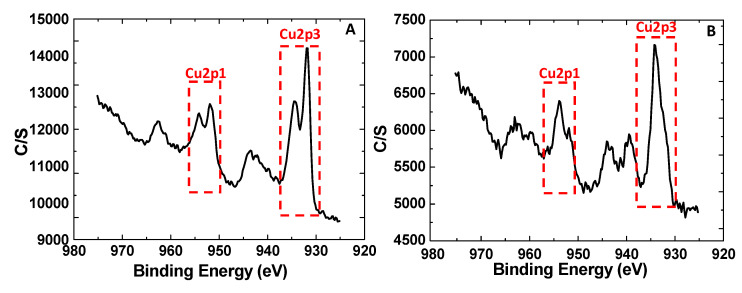
The X-ray photoelectron spectra for (**A**): Cu(I/II)BTC after the reduction of Cu(II)BTC; (**B**) the starting Cu(II)BTC material synthesized at 140 °C and 180 min of reaction.

XPS was carried out to investigate the oxidation state of Cu(II)BTC and after its reduction. The XPS and the fitted peaks in the Cu 2p_3/2_ region are shown in Figure 4. The peak at 935 eV is attributed to Cu(II) in the Cu(II)BTC. After the partial reduction of Cu(II)BTC to Cu(I)BTC, an additional peak at 933 eV corresponding to Cu(I)BTC can be easily identified. The deconvolution of the two peaks yields a Cu(II):Cu(I) ratio of 42.2:57.8 in the reduced Cu(I/II)BTC. Thermal stability of the synthesized MOF particles are shown in Figure S4 of the Supplementary Document. In the descriptions below, Cu(I/II)BTC will be used to indicate this partially reduced MOF.

### 3.2. Adsorption Experiments

**Figure 5:**
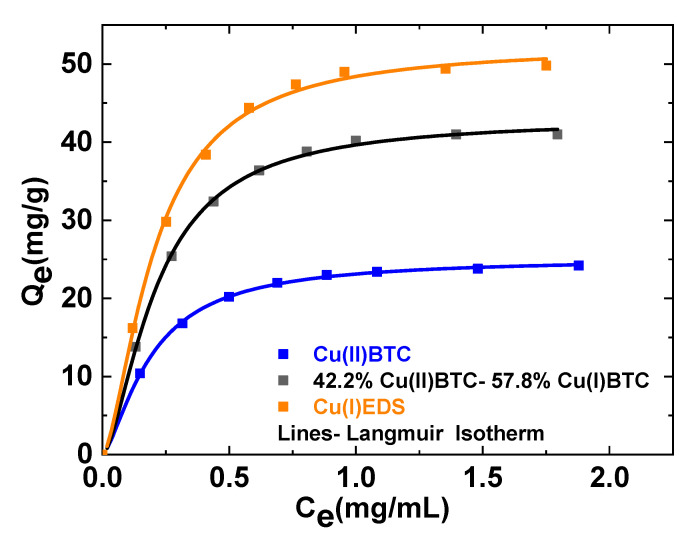
Langmuir adsorption isotherms of toluene for membranes incorporating 7 wt% Cu(II)BTC, Cu(I/II)BTC and Cu(I)EDS MOFs respectively at 298 K after 24 h of membrane equilibration with toluene/n-heptane solvent mixtures for the three oxidation states of the MOFs incorporated.

To understand the affinity of solvents towards the nanocomposite membrane, static adsorption experiments with different concentrations of toluene for membrane containing 7 wt% MOFs have been carried out, and the behavior is shown in Figure 5. Fabricated MMMs were equilibrated with toluene/n-heptane feed mixtures containing various concentrations of toluene for about 24 h. The details of the adsorption experiments have been described in the previous section. The static adsorption experiments are the best indication of the affinity of the MOFs towards toluene as well as the equilibrium behavior of MOFs at different toluene concentration. The Langmuir isotherm was used to evaluate the maximum adsorption capacity of the MOFs.

MMMs with Cu(I)EDS have the highest toluene adsorption capacity, followed by the membranes with the mixed-valence state, Cu(I/II)BTC. Membranes with the highest oxidation state Cu(II)BTC have the lowest adsorption capacity. The maximum adsorption capacities are 52.4, 44.0, and 25.2 mg/g of MOF for membranes incorporating Cu(I)EDS, Cu(I/II)BTC, and Cu(II)BTC, respectively. Some other parameters (membrane structure) may also play an important role during solvent adsorption including the size, crystallinity and the linker of the MOFs incorporated as well as the membrane porosity [52,53].

### 3.3. Membrane Characterization

**Figure 6:**
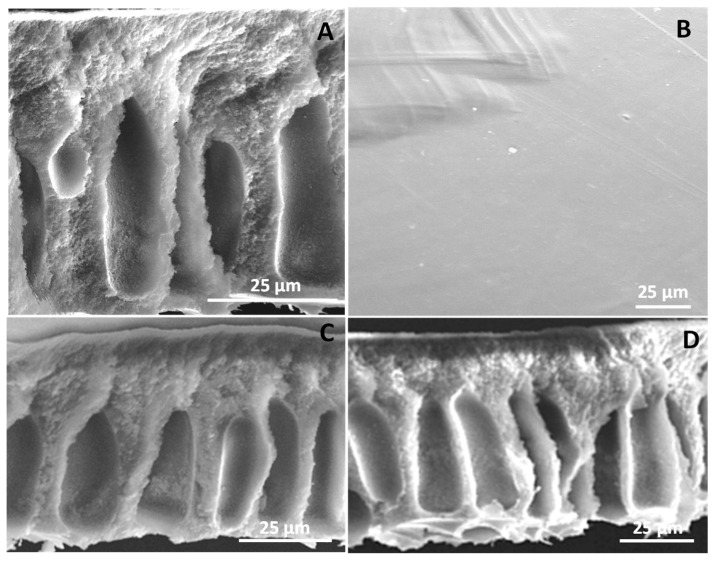
SEM images of mixed-matrix membranes (MMMs) fabricated: (**A**) cross-section of MMM containing Cu(II)BTC, (**B**) top surface of MMM containing Cu(II)BTC, (**C**) cross-section of MMM containing Cu(I/II)BTC, and (**D**) cross-section of MMM containing Cu(I)EDS. All membranes were fabricated using a mixed solvent with DMF:1,4-dioxane ratio of 3:1.

Figure 6 shows the SEM images of membrane cross-sections containing copper-based MOFs. All the membranes contain about 3 wt% MOFs. The membrane fabrication was initially carried out with a 1:1 ratio of DMF to 1,4-dioxane. However, a rather dense structure was formed. Thereafter, it was found that a solvent composition of 3:1 for the DMF:1,4-dioxane resulted in the formation of macrovoids during phase separation leading to finger-like projection across the cross-section [54]. The preparation of casting solution with MOFs was one of the challenging processes. As the dosage of MOFs increases, the solution viscosity also increases, which makes it difficult to mix well to obtain a homogeneous solution.

The thickness of the membranes varies with the amounts of MOFs incorporated. The membrane with 3 wt% of Cu(II)BTC has a thickness of 55 µm, whereas it increases to 63 µm when partially reduced 3 wt% Cu(I/II)BTC was incorporated in the casting solution. Membrane thickness further increases to 68 µm when 3 wt% Cu(I)EDS was incorporated. A similar trend was observed for the top skin layer. The skin layer thickness for membranes with Cu(II)BTC, Cu(I/II)BTC, and Cu(I)EDS were found to be 1.25, 1.9, and 2.3 µm, respectively [53]. Membrane formation is rather complex and often affected by the small differences in additives in the casting solution.

### 3.4. Separation Experiments with n-Heptane/Toluene System

Previously membrane-based pervaporation process was investigated extensively for the separation of n-heptane and toluene [2,11,12,13,14,15,16,17,18,19,20,21,35,36]. Pervaporation takes advantage of the different solubility and diffusivity of the two compounds in the vapor phase through membrane materials to achieve the separation. An elevated feed temperature has to be used in order to increase the vapor pressure of the liquid mixture. Since toluene is an aromatic compound, it has enhanced interaction and diffusion through the dense pervaporation membranes. However, due to the similarity of the two compounds in their boiling points and sizes, separation factors achieved remain relatively moderate ranging from about 3 to 18. Moreover, the flux measured varies from 30 to 4600 gm^−2^h^−1^ depending on the temperature of the feed and membrane materials used. At temperature of 40 °C, the fluxes obtained are extremely low with less than 100 gm^−2^h^−1^ for most of the membranes. The main challenge for pervaporation and many other membrane processes is the trade-off between flux and separation performance, which made it hard for further improvement and for industrial application. Moreover, solvent swelling of the membrane materials is another major issue which leads to the degradation of the separation performance.

Previously, temperature was considered as one of the important parameters to enhance the vapor phase concentration to separate the aprotic solvents like n-heptane and toluene mixture. Here a liquid phase process based on nanofiltration was adopted while keeping the temperature constant. Pressure of up to 180 psi was applied during the filtration experiments. The driving force for separating toluene and n-heptane was based on their relative affinity to the MOFs incorporated and their relative mobility in the membrane. Here PI membranes incorporating different oxidation states of Cu MOFs including Cu(II)BTC, Cu(II/I)BTC and Cu(I)EDS were used as discussed previously.

**Figure 7:**
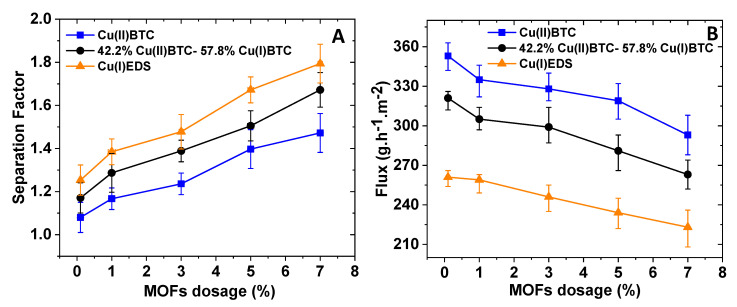
Separation factor (**A**) and flux (**B**) for mixed-matrix membranes (MMMs) with different percentages of Cu(II)BTC, Cu(II/I)BTC, and Cu(I)EDS for the separation of toluene and n-heptane from a feed mixture with 58 mol% of toluene.

Figure 7 shows the separation factors (Figure 7A) and fluxes (Figure 7B) for MMMs incorporating different amounts of MOFs for the feed containing 58:42 (mol%) of toluene and n-heptane. During the filtration experiments with the PI support only and without MOF particles, the membrane didn’t show any selectivity between toluene and n-heptane. However, when MOF percentages in the range of 0.1–7 wt% were incorporated in the membranes, a preferred selectivity of toluene over n-heptane was shown. It can be seen from Figure 7A that the higher the MOF loading in the membrane, the higher the separation factor. Moreover, the oxidation state of the Cu ion also has a strong impact on the separation factor. It appears that the Cu(I)EDS has the highest selectivity over toluene, followed by Cu(I/II)BTC. Cu(II)BTC has the lowest separation factor. The separation factors are only around 1.08 when only 0.1 wt% of the Cu MOFs incorporated in the membrane. However, the separation factors reach 1.47, 1.67 and 1.79 when 7 wt% of Cu(II)BTC, Cu(I/II)BTC and Cu(I)EDS were incorporated in the membranes respectively.

The flux shows the opposite trend varying from 353 to 293 gm^−2^h^−1^ when the amount of Cu(II)BTC increased to 7 wt% from 0.1 wt%. The value further declined to 223 gm^−2^h^−1^ when 7 wt% Cu(I)EDS was incorporated, which exhibited the highest separation factor in the present studies. During long-term testing, the membranes with a mixed-valence state of copper showed a decline in separation factor, which could be due to the oxidation of Cu(I) in the mixed state, whereas the membranes with complete (II) and (I) state did not show any changes in separation efficiency.

Based on the static adsorption and dynamic separation studies, it is clear that the affinity interaction between the MOF particles incorporated and the aromatic toluene is the major driving force for separating toluene from heptane. Since the affinity interaction is based on cation–π and π–π interactions between the copper ion and toluene or between aromatic BTC with toluene, the oxidation state of the Cu ion is expected to affect the cation–π interaction as was observed experimentally here. It is expected that divalent Cu^2+^ ion has a stronger interaction with the same aromatic compound than the monovalent Cu^+^ ion. However, membranes incorporating Cu(II)BTC have the lowest separation factors among the three Cu MOFs investigated. This is probably due to the fact that membranes incorporating Cu(II)BTC are the thinnest in membrane thickness and lowest in skin layer depth whereas the corresponding membranes incorporating Cu(I)EDS have the thickest membrane layer and largest skin layer. The differences in separation factors observed can result partially from the differences in macroscopic physical properties and overall barrier layer thickness. In addition, it is known that Cu(II)BTC, Cu(I/II)BTC and Cu(I)EDS have different crystal lattice and symmetry. Cu(II)BTC has a cubic crystal lattice with *Fm*$\bar{3}$*m* symmetry [55]. The mixed valence Cu(I/II)BTC consists of two interpenetrating three-dimensional nets of Cu(II) dimers and tetrameric Cu(I) forming a complex framework [46]. Cu(I)EDS has a triclinic crystal lattice with P$\bar{1}$ symmetry [47]. Because of the pore structures are drastically different, it is expected that the interactions between toluene and the MOF particles with different oxidation states and the resulting adsorption capacities are also rather different.

Along with cationic–π and π–π interactions, the temperature and the pressure applied on the upstream side played do affect both separation factor and flux. Another critical factor is the thickness of the entire membrane, as well as the active layer. Figure 6 shows the SEM images of membranes with 3 wt% of MOFs, where thickness varied from 55 to 68 µm when Cu(II) BTC were replaced with Cu(I)EDS. The increased thickness improves the separation factor.

### 3.5. Swelling Studies

**Figure 8:**
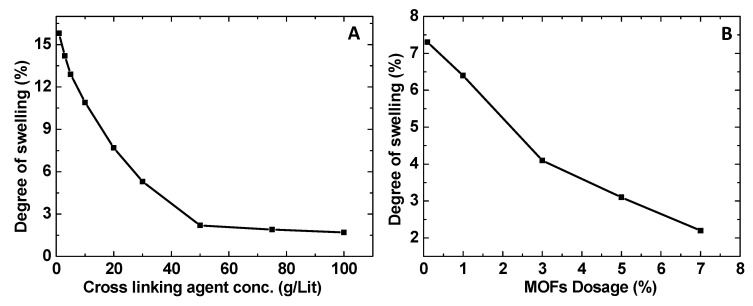
(**A**) Variation of degree of swelling as a function of crosslinking agent concentration for membranes incorporating 7 wt% Cu(II)BTC; (**B**) variation of degree of swelling as the percentage of MOFs incorporated in the membrane which was treated with 50 g/L of the crosslinking agent.

Figure 8 shows the swelling behavior of the membranes fabricated with different concentrations of crosslinking agent (A) and MOF dosage (B). Processing the fabricated membrane with a crosslinking agent is essential to improve its mechanical strength and solvent resistance. As shown in Figure 8A, the degree of swelling decreases with the increase of the crosslinking agent concentration. The degree of swelling is above 15% without crosslinking. It decreases rapidly to about 3% when the crosslinking agent concentration increases to about 40 g/L.

Further increase in the crosslinking agent only slightly improves the swelling reaching a minimum value of 1.7% observed at 100 g/L of crosslinking agent. The amounts of MOF incorporated in the membranes also significantly affects membrane swelling, as shown in Figure 8B. The degree of swelling decreases from 7.3 to 2.2% when the percentage of MOF increased from 0.1 to 7 wt%. Similar behaviors were observed for all three MOFs with different oxidation states since their particle sizes are similar.

It was further observed that as the concentration of crosslinking agent increases, the time needed for permeate to appear also increases. The higher the degree of swelling, the higher the membrane flux due to the increased fractional free volume in the hybrid membrane. This leads to the degradation of the separation performance. The performance of these membranes is compared with the state-of-the-art research in the same filed and depicted in Table 2. Table 2 lists the separation factors and fluxes from previous studies [2,11,12,13,14,15,16,17,18,19,20,21,35,36] with pervaporation for separating toluene from n-heptane. The separation factors range from just above 3 to almost 18. Fluxes range from 50 to 590 gm^−2^h^−1^. Temperatures used for these pervaporation processes range from 40 to 85 °C. For pervaporation experiments performed at 40 °C, measured fluxes are all very low with less than 100 gm^−2^h^−1^ except one case reaching 270 gm^−2^h^−1^. One previous study [16] used Cu(II)BTC and polyvinyl alcohol forming nanohybrid membrane for the pervaporation separation of toluene from n-heptane at 45 °C. The membrane exhibited a separation factor of 17.9 for toluene over n-heptane and a flux of 150 gm^−2^h^−1^. As temperature increases, generally flux also increases reaching close to 500 gm^−2^h^−1^ at 80 °C. Higher temperature will increase the diffusion and solubility of both toluene and n-heptane in the membrane as well as increase the mobility and fractional free volume of the polymers. This increased solubility and mobility will increase the pervaporation flux as was shown in Table 2. However, there is no clear correlation between separation factor and temperature for the pervaporation separation of toluene and n-heptane.

As also listed in Table 2, our liquid phase nanofiltration process of separating toluene from n-heptane has several distinctive features compared to previous pervaporation studies. Firstly, it is a room temperature process that does not require high energy input to heat the feed streams. Secondly, the flux for the current nanofiltration process is about 10 times higher than the most of the pervaporation processes at 40 °C. Thirdly, the separation factors from this study can reach over 3, comparable to some of the pervaporation processes. Finally, our results demonstrate that it is possible to further increase the separation performance of our mixed matrix membranes by increasing the percentage of the MOF materials incorporated or manipulating the adsorption/desorption energetics. Optimizing the adsorption/desorption processes could involve using different types of transition metal ions, or even some functional organic compounds which have stronger affinity with the aromatic compounds for separating aliphatic and aromatic mixtures.

## 4. Conclusions

In summary, we have successfully synthesized modulator-assisted copper-based MOF particles using a simple stainless-steel reactor in a sand bath achieving controllable nanoparticles sizes. Three different types of copper-based MOFs have been synthesized and incorporated into the polyimide matrix forming mixed matrix membranes with different doses of MOF. The membranes were tested for n-heptane and toluene separation by applying the pressure in a specific stepwise mode at room temperature. The valence state of the MOF and membrane structure are found to play a pivotal role in the separation of the two compounds. Membranes with a mixed-valence state Cu(I/II)BTC exhibit a higher separation factor of 1.47 compared to 1.67 from Cu(II)BTC. The efficiency further enhances to 1.79 when Cu(I)EDS particles were incorporated in the membrane. The cationic–π and π–π interactions between toluene and incorporated MOFs in the mixed matrix membranes, and dosage of MOFs lead to the selectivity of toluene over n-heptane. The swelling of polymeric membranes has been reduced to 2.2% as MOFs dosage increases to 7 wt%. Both MOFs and cross-linking agent concentration play a critical role in the swelling of the membrane and thereby, the separation factor.

## Figures and Tables

**Table 1:** Structural properties of synthesized MOFs.

Properties	Cu(II)BTC	Cu(I/II)BTC	Cu(I)EDS
Particle size (nm)	25.1	24.9	22.6
BET surface area (m^2^g^−1^)	1123.7	1069.2	983.4

**Table 2:** Comparison of the present study with previously published studies for separation of toluene/n-heptane mixture.

Membranes	Temperature (°C)	Toluene Concentration in the Feed	Flux (g·m^−2^·h^−1^)	Separation Factor
Polyurethanes cross-linked with PEGHT (polyethylene glycol-isocyanate-2,2′,2”-nitrilotriethanol) [20]	50	50 wt%	1700	9.0
	80	20 wt%	4600	8.0
Poly (oxyethylene 360 methacrylates) /poly(acrylonitrile) [21]	80	20 wt%	330	6.6
Poly (oxyethylene 400 methacrylates) /poly(acrylonitrile) [21]	80	20 wt%	1270	6.7
Cellulosic ester - polyethylene glycol-600-dimethacrylate [22]	80	20 wt%	580	8.9
2,2-bis(3,4-dicarboxyphenyl) hexafluoro propane dianhydride/2,2-bis [4-(4-amino phenoxy) phenyl] hexafluoro propane/Polyimide [19]	80	20 wt%	760	6.5
PolyAn modified aromatic selective polyvinylchloride [18]	75	40 wt%	230	6.0
Sulfoethyl cellulose/benzyl dodecyl dimethylammonium chloride [36]	80	20 wt%	740	3.4
Methyl methacrylate-co-methacrylic acid (3-sulfopropyl ester) potassium salt/benzyl dodecyl dimethylammonium chloride [36]	80	20 wt%	2190	3.5
Surface modified polyacrylonitrile (PolyAn) [18]	45	40 wt%	3920	3.26
	85	20 wt%	5900	3.63
Cu(II)BTC/poly (vinyl alcohol) tubular membrane [17]	45	50 wt%	150	17.9
Aromatic polyimide and polybenzoxazole [2]	80	40 wt%	15–46	4.7
Poly(γ-benzyl-l-glutamate) (PBG) on a micro-porous poly(amide-imide) [15]	50	40 wt%	320	3.8
Poly(ethylene glycol) methacrylate (PEO526OHMA)- poly(acrylonitrile) [16]	80	20 wt%	1860	7.8
Poly(vinyl alcohol)-graphene oxide [12]	40	50 wt%	27	12.9
Polyvinylchloride/Nanocor clay [14]	74	50 wt%	690	4.0
Poly(ether-*block*-amide) (PEBA) [3]	40	50 wt%	65	4.3
Poly(vinyl chloride) (PVC)–activated carbon (Maxsorb SPD30) [34]	74	50 wt%	90	6.3
Polyimide + 7% Cu(II)BTC [This work)	35	58 mol%	293	1.47
Polyimide + 7% [42.2% Cu(II)BTC-57.8% Cu(I)BTC [This work]	35	58 mol%	263	1.67
Polyimide + 7% Cu(I)EDS [This work]	35	58 mol%	223	1.79

## Supplementary Materials

The following are available online at https://www.mdpi.com/2077-0375/10/11/313/s1.
