# Supplementary material for: Cu(I/II) Metal–Organic Frameworks Incorporated Nanofiltration Membranes for Organic Solvent Separation
Source: Membranes (Basel). 2020 Oct 29;10(11):313. doi: 10.3390/membranes10110313 (PMC7692870; doi:10.3390/membranes10110313)
Supplement: Supplementary file 1 [file membranes-10-00313-s001.pdf]

SUPPORTING INFORMATION

Cu(I/II) MOF Incorporated Nanofiltration Membranes for Organic Solvent Separation

Lakshmeesha Upadhyaya [1,2], Yu-Hsuan Chiao [3], S. Ranil Wickramasinghe [3,*] and Xianghong Qian [1,*]

[1] Department of Biomedical Engineering, University of Arkansas, Fayetteville, Arkansas 72701.

[2] King Abdullah University of Science and Technology (KAUST), Biological and Environmental Science and Engineering Division (BESE), Advanced Membranes and Porous Materials Center (AMPM), 23955-6900 Thuwal, Saudi Arabia

[3] Ralph E Martin Department of Chemical Engineering, University of Arkansas, Fayetteville, Arkansas 72701

* Correspondence: xqian@uark.edu (X.Q.); swickram@uark.edu (S.R.W.)

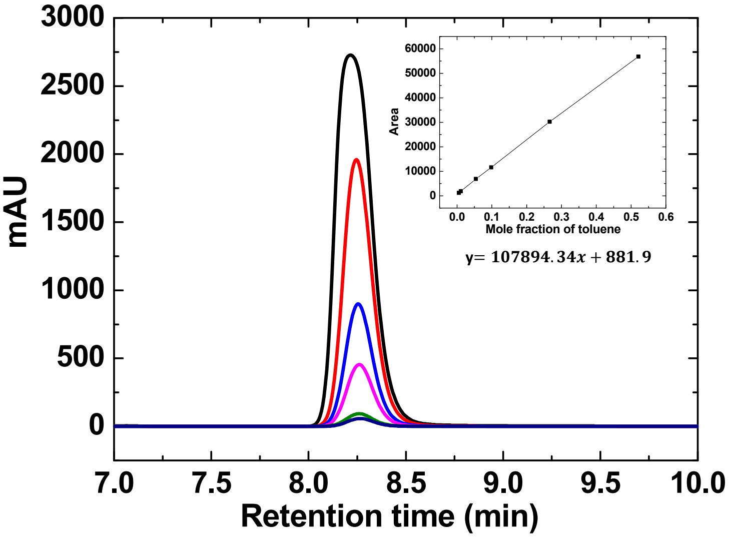

Figure S1. Analysis of different concentration of toluene through HPLC at wavelength of 254 nm. The inset shows the calibration curve (Area v/s molar fraction of toluene).

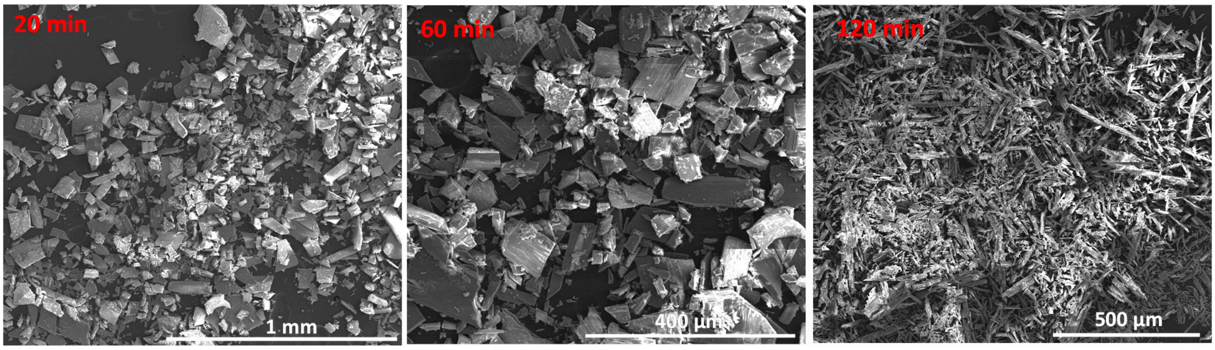

Figure S2. SEM images of Cu(II)BTC synthesis using DDA as a modulator using autoclave technique and different time interval.

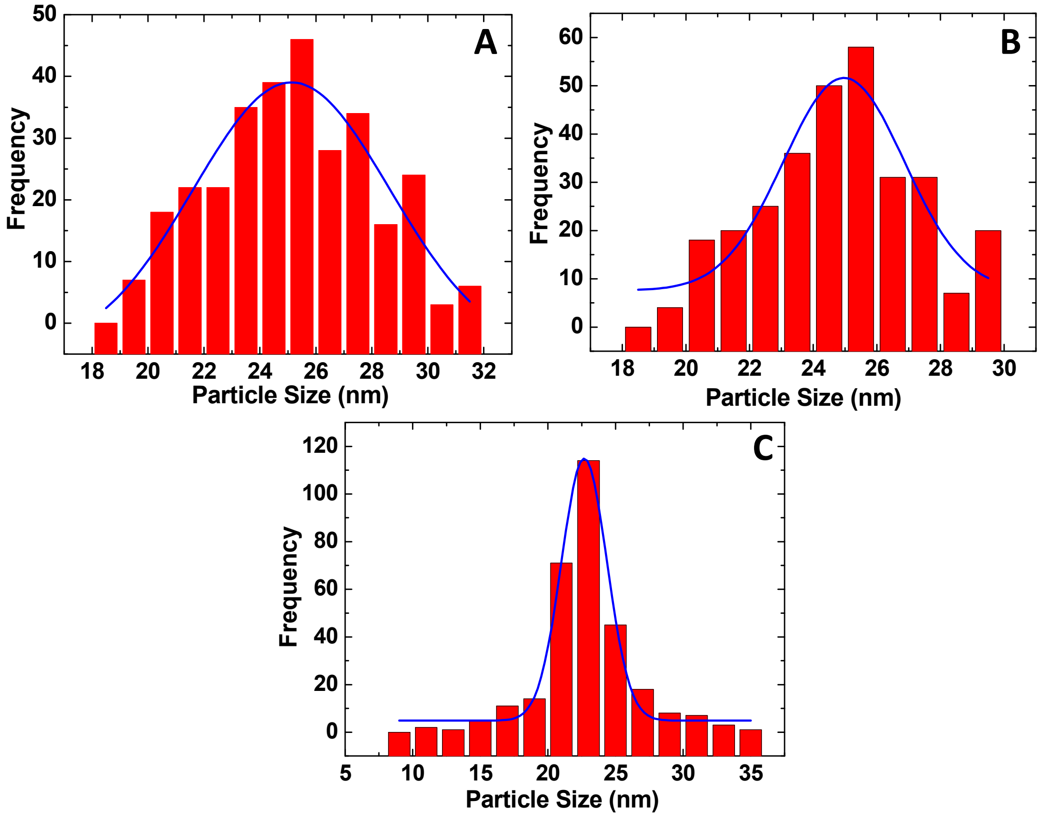

Figure S3. Particle size calculated from SEM images using ImageJ software for (A) Cu(II)BTC, (B) Cu(II/I) BTC and (C) Cu(I)EDS.

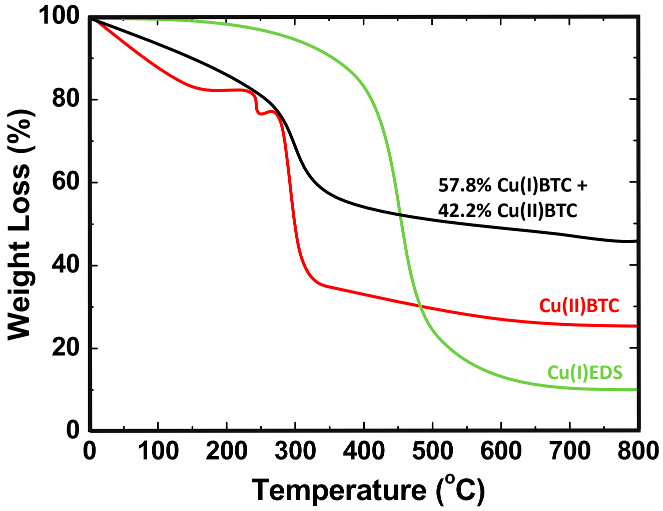

Figure S4. Thermogravimetric analysis of three different types of MOFs
